# Hydrogel-Enhanced Autologous Chondrocyte Implantation for Cartilage Regeneration—An Update on Preclinical Studies

**DOI:** 10.3390/bioengineering11111164

**Published:** 2024-11-19

**Authors:** Xenab Ahmadpoor, Jessie Sun, Nerone Douglas, Weimin Zhu, Hang Lin

**Affiliations:** 1 Department of Orthopaedic Surgery, University of Pittsburgh School of Medicine, 450 Technology Drive, Rm 217, Pittsburgh, PA 15219, USA; xea1@pitt.edu (X.A.); jes295@pitt.edu (J.S.); 2 Department of Molecular Oncology, University of Pittsburgh School of Medicine, 450 Technology Drive, Rm 217, Pittsburgh, PA 15219, USA; douglas.neronekiyoshiomari@medstudent.pitt.edu; 3 Department of Sports Medicine, The First Affiliated Hospital of Shenzhen University, Shenzhen Second People’s Hospital, Shenzhen 518025, China; 4 Clinical College of the Second Shenzhen Hospital, Anhui Medical University, Shenzhen 518025, China; 5 Department of Bioengineering, University of Pittsburgh Swanson School of Engineering, 450 Technology Drive, Rm 217, Pittsburgh, PA 15219, USA; 6 Orland Bethel Family Musculoskeletal Research Center, University of Pittsburgh, Pittsburgh, PA 15260, USA

**Keywords:** fixation, integration, cartilage, ACI, hydrogel

## Abstract

Autologous chondrocyte implantation (ACI) and matrix-induced ACI (MACI) have demonstrated improved clinical outcomes and reduced revision rates for treating osteochondral and chondral defects. However, their ability to achieve lasting, fully functional repair remains limited. To overcome these challenges, scaffold-enhanced ACI, particularly utilizing hydrogel-based biomaterials, has emerged as an innovative strategy. These biomaterials are intended to mimic the biological composition, structural organization, and biomechanical properties of native articular cartilage. This review aims to provide comprehensive and up-to-date information on advancements in hydrogel-enhanced ACI from the past decade. We begin with a brief introduction to cartilage biology, mechanisms of cartilage injury, and the evolution of surgical techniques, particularly looking at ACI. Subsequently, we review the diversity of hydrogel scaffolds currently undergoing development and evaluation in preclinical studies for articular cartilage regeneration, emphasizing chondrocyte-laden hydrogels applicable to ACI. Finally, we address the key challenges impeding effective clinical translation, with particular attention to issues surrounding fixation and integration, aiming to inform and guide the future progression of tissue engineering strategies.

## 1. Introduction

Hyaline cartilage, the most prevalent form of cartilage in the body, is commonly found in vital areas such as between costal ribs, within the respiratory system (trachea), and on the surface of the bone, referred to as articular cartilage [1]. This specialized connective tissue plays a crucial role in ensuring a seamless and well-lubricated surface, effectively minimizing friction, and facilitating the transmission of loads to the underlying subchondral bone. Due to a variety of factors, including person-level factors (e.g., age, sex, health), environmental factors (e.g., occupational and physical activity), and joint level factors (e.g., bone/joint shape, muscle strength), articular cartilage undergoes wear and tear over time [2]. This makes this connective tissue vulnerable to focal defects triggered by mechanical injuries or trauma. Without prompt and appropriate interventions, focal cartilage defects are often debilitating and cause degradation of surrounding tissues. Clinically, cartilage injuries have been shown to be associated with an increased risk of osteoarthritis and are predictive of total joint arthroplasty [3].

Effectively addressing articular cartilage defects in different joints poses a considerable challenge, primarily due to its avascular nature. The lack of direct blood supply subsequently impedes natural tissue regeneration mechanisms reliant on recruiting stem/progenitor cells to the damaged site [4]. Current early intervention strategies tend to favor conservative treatments that aim to slow the progression of cartilage degradation, such as wearing a brace, taking non-steroidal anti-inflammatory medications (NSAIDs), and receiving corticosteroid injections [5]. While these approaches may alleviate symptoms, clinical studies indicate their inability to promote the regeneration of new tissue that mirrors the zonal structure and unique functional properties found in native articular cartilage [6].

As articular cartilage defects progress, surgical techniques, such as abrasion arthroplasty, microfracture, and transplantation of osteochondral plugs (both autografts and allografts), are often employed to restore normal joint congruity and mitigate further joint deterioration. Despite their widespread use, these surgical methods frequently fail to provide long-term solutions for articular cartilage defects [7]. Consequently, contemporary research increasingly focuses on investigating regenerative medicine and tissue engineering approaches. In this context, hydrogels loaded with chondrocytes or progenitor cells have emerged as a particularly noteworthy area of interest [8]. These malleable three-dimensional (3D) polymer networks can mimic the properties of natural extracellular matrices (ECMs), making them versatile and appealing biomaterials for applications in tissue engineering and cell therapy. When utilized as cell-seeded tissue engineering scaffolds, hydrogels have demonstrated their utility in facilitating chondrocyte attachment, maintaining chondrocyte phenotype, and transferring loads to the chondrocytes [9].

Despite significant advances in modifying and improving ACI, crucial obstacles persist in the clinical application of implants, such as the lack of hydrogels that fully simulate the mechanical properties of hyaline cartilage and the limited integration of newly formed constructs with the surrounding tissue [10]. These challenges directly influence the stability of regenerated cartilage and, consequently, the ability to perform functions similarly to native tissue. Therefore, the main objective of this review is to provide a concise and comprehensive summary of hydrogel scaffolds that have been used in ACI, with a focus on those that have been tested in preclinical studies. We aim to illuminate notable developments in the field in the past 10 years, discussing both their potential and limitations in effectively integrating with native cartilage and how this impacts their use in clinical applications.

## 2. Articular Cartilage

### 2.1. Articular Cartilage Composition and Phases

Articular cartilage, a crucial component of joint function, is characterized by two integral phases: the solid phase and the liquid phase. The solid phase encompasses collagen fibers, proteoglycans, glycosaminoglycans, and glycoproteins, collectively shaping the tissue’s overall structure [11]. The liquid phase consists of water and electrolytes, establishing the solid components’ environment. Together, these phases determine cartilage’s biomechanical properties [12].

### 2.2. Chondrocyte Function and Zonal Classification

Chondrocytes, the major cell type in the cartilage, play a pivotal role in producing and maintaining the extracellular matrix (ECM) by synthesizing type II collagen, proteoglycans, and enzymes. Despite being classified as a singular cell type, variations in their morphology, density, and organization contribute to the heterogeneity of cartilage [13]. This diversity leads to the characterization of articular cartilage into four distinct zones: the superficial (tangential) zone, middle (transitional) zone, deep (radial) zone, and calcified zone.

The superficial zone is characterized by its contact with the synovial fluid and its high concentration of flattened chondrocytes. It is responsible for most of the tensile properties of cartilage, enabling it to resist the shear, tensile, and compressive stresses imposed by the articulation of joints [14]. The collagen fibers of this zone (primarily type II collagen) are packed tightly and aligned parallel to the articular surface. The transitional zone, housing more spherical chondrocytes, serves as the initial defense against compressive forces. Constituting 40–60% of the total cartilage volume, this zone contains proteoglycans and thicker collagen fibrils organized obliquely [15]. In the deep zone, chondrocytes adopt a columnar orientation, and collagen fibrils are arranged perpendicular to the articular surface. This zone, rich in proteoglycan concentration, provides the highest resistance to compressive forces [15]. Finally, the calcified layer assumes a crucial role in ensuring cartilage integrity by anchoring the collagen fibrils of the deep zone to the subchondral bone. This intricate organization and composition collectively contribute to the overall functionality and resilience of articular cartilage in joint biomechanics.

### 2.3. Matrix Classification

Beyond the zonal classification, the articular cartilage matrix undergoes further categorization into three distinct regions: pericellular, territorial, and interterritorial [13], depending on their distance to the residing chondrocytes. The pericellular matrix, a thin layer adjacent to the cell membrane, primarily contains proteoglycans, collagen type VI, along with glycoproteins and other non-collagenous proteins. This matrix is believed to serve a role in initiating signal transduction within cartilage under load-bearing conditions [16]. The territorial matrix envelops the pericellular matrix, characterized by fine collagen fibrils that intricately form a network around the cells. Thicker than the pericellular matrix, the territorial matrix is postulated to provide protection to cartilage cells against mechanical stresses, enhancing their ability to withstand substantial loads. The interterritorial region, the largest among the three matrix regions, significantly contributes to the biomechanical properties of articular cartilage. This intricate division into pericellular, territorial, and interterritorial regions further elucidates the complexity and functional specialization within the microarchitecture of articular cartilage.

## 3. Cartilage Injury

**Figure 1:**
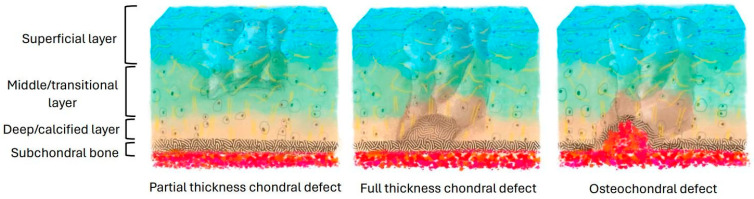
Different types of cartilage injury.

The common types of cartilage injury include (1) superficial and middle zone lesions, (2) lesions reaching the subchondral bone without penetrating the marrow, and (3) lesions penetrating the marrow [11] (Figure 1). Acute or repetitive blunt trauma, such as excessive loading, can damage the cellular membrane, resulting in an efflux of intracellular contents that causes a decrease in the metabolic activity of the cell [17]. This alteration of the matrix results in decreased proteoglycan concentration and potential collagen fibril framework disruptions. During this early stage of chondrocyte injury, chondrocytes possess the ability to detect changes in the matrix composition and synthesize new molecules to facilitate the repair of the macromolecular framework [18]. However, if the newly synthesized matrix and proliferating cells fail to fill the tissue defect adequately, the heightened proliferative and synthetic activity halts soon after injury [19].

When injury extends into the subchondral bone, it induces hemorrhage and fibrin clot formation, triggering an inflammatory response [20]. Blood escaping from the damaged bone vessels initially forms a hematoma, temporarily occupying the injury site. Platelets within the clot then release vasoactive mediators and growth factors, stimulating vascular invasion and migration of undifferentiated cells, such as mesenchymal cells, into the clot. These cells proliferate and synthesize into a new matrix that is composed of a higher ratio of collagen type I and II and less proteoglycan, making it morphologically and mechanically inferior to the original native articular cartilage tissue [21]. The inherent biomechanical weakness of fibrocartilaginous tissue leads to discontinuities, full-thickness fissures, and loss of repair tissue [22].

## 4. Current Treatments of Cartilage Defects

### 4.1. Initial Assessment

While not symptomatic in all patients, articular cartilage lesions have the potential to mimic the severity and limitations of end-stage osteoarthritis of the knee [23]. The diagnosis process begins with the evaluation of clinical symptoms, followed by a thorough joint examination utilizing imaging techniques, and concludes with a definitive evaluation through arthroscopy. Given the varied degree of symptoms associated with cartilage defects, a physical examination aims to identify any factors hindering cartilage restoration and to determine the necessity for additional procedures. This examination includes assessing standing and gait and evaluating rotational stability, malalignment, flexibility, and muscle weakness [24]. Following the physical examination, imaging is utilized to characterize any cartilage lesions, ligament, and meniscus injuries. Knee radiographs also enable evaluation for fractures, loose bodies, osteophytes, joint space narrowing, and mechanical axis alignment.

In suspected cases of bone injury, computed tomography (CT) provides detailed anatomical information on subchondral bone. As cartilage damage progresses, morphological magnetic resonance imaging (MRI) sequences may reveal surface fibrillation and erosions, with the irregular superficial surface of cartilage contrasting sharply against the adjacent synovial fluid [25]. Subsequently, arthroscopic assessment can be conducted to comprehensively evaluate the knee joint, such as examining the menisci for tears or prior resection and inspecting articular surfaces for defects [26]. To standardize severity assessments in clinical settings, grading scales based on clinical findings have been developed to characterize cartilage lesions, such as the Outerbridge classification system that categorizes chondral lesions into four grades without specifying lesion depth [27]. The most used scale by imagers is the International Cartilage Repair Society (ICRS) system, which grades based on lesion depth and the involvement of subchondral bone [28] (Table 1).

### 4.2. Non-Surgical Interventions

After assessing the severity of the lesion, the subsequent step involves considering a variety of interventions, both conservative and surgical, to address the issue. Conservative, non-operative therapies aim to manage symptoms and control disability, potentially slowing down articular cartilage breakdown [29]. These conservative treatments encompass a range of non-pharmacological interventions (e.g., physical therapy) and pharmacological treatments (e.g., NSAIDs), both of which have demonstrated mixed success [30]. The effects of these therapies include reduction of pain, improvement in cartilage function and thickness, and improvement in the anti-inflammatory and antioxidant responses [31]. However, in many instances, these treatments alone are insufficient to restore cartilage [32], and surgical interventions become necessary. This is particularly observed in young, active patients with symptomatic full-thickness chondral lesions [33].

### 4.3. Surgical Interventions

When surgical intervention is required, four primary procedures are commonly employed [34]: chondroplasty, marrow stimulation (typically via microfracture), osteochondral restoration (including osteochondral autograft transfer and osteochondral allograft transfer), and cell-based repair techniques such as autologous chondrocyte implantation and matrix-assisted autologous chondrocyte implantation. Chondroplasty and microfracture procedures constitute the primary interventions for treating articular cartilage issues in the knee. Yet, these options come with notable limitations. Chondroplasty, typically reserved for minor wear [35], has demonstrated potentially subpar long-term outcomes [36]. In microfracture, the stimulation of cartilage regeneration involves inducing stem migration through perforations in the underlying bone matrix [37]. However, this method yields fibrocartilaginous tissue, which lacks the structural integrity of native hyaline cartilage [38]. Studies have revealed that between 47 and 80% of patients experience diminished functional outcomes beyond the 24-month postoperative period [39]. Conversely, mosaicplasty utilizing osteochondral auto/allografts has shown promise with superior clinical results compared to microfracture [40,41]. Nonetheless, challenges such as graft fragmentation and collapse contribute to failure rates ranging from 4 to 21% at 35–48-month follow-up and 15 to 39% after 7 years of follow-up [42]. Moreover, mosaicplasty with autografts is restricted to lesions smaller than 4 cm2 to minimize donor site morbidity. While fresh allografts could address larger defects, their availability is limited [37,43].

## 5. ACI-Based Therapy

Initially introduced by Brittberg and Peterson in 1987 in Europe, ACI involves harvesting chondrocytes from cartilage biopsy samples and culturing them in vitro [44]. Subsequently, the cell suspension is implanted into the cartilage defect and covered with a cap made from periosteum tissue (ACI-P) [45]. ACI utilizes autologous cells to regenerate cartilage tissues, which offers numerous advantages over traditional techniques. Unlike allogeneic grafting methods, ACI reduces the risk of immune rejection and facilitates a more natural integration with existing tissue [46]. ACI is also expected to promote the regeneration of hyaline-like cartilage, closely resembling native tissue, leading to improved long-term outcomes and decreased risk of degeneration [47]. These characteristics position ACI as a promising intervention, particularly for patients with larger or more complex cartilage defects where traditional methods may be less effective. For example, long-term case series with over 10 years of follow-up have demonstrated that ACI is an effective and durable treatment for large (>4 cm^2^) knee cartilage defects [48].

However, complications such as overgrowth (hypertrophy) and scarring (arthrofibrosis) at the implantation site have occurred after ACI and often require further surgery [49]. To overcome these challenges, subsequent generations of ACI have been developed. The second generation replaced the periosteal patch with a biodegradable collagen membrane known as ACI-C. Although this mitigated the drawbacks of the periosteal cover, it introduced complications associated with suturing the collagen cover, including tissue microtrauma and cell leakage. In the third generation of ACI, chondrocytes were delivered on a pre-seeded collagen membrane known as matrix-induced autologous chondrocyte implantation, or MACI. The membrane is sized according to a template and then placed with the porous side facing the subchondral bone and secured with fibrin glue and sutures. These patches can be implanted using a less invasive surgical approach, requiring less operative time than ACI-C [46]. Importantly, MACI has gained clearance from the U.S. Food and Drug Administration (FDA), marking a significant advancement in ACI technology.

## 6. Hydrogels in ACI

In the last few decades, researchers have been exploring various biomaterials for cartilage tissue engineering applications, marking a rapidly evolving field that employs different cell types, biodegradable scaffolds, bioactive agents, and physical stimuli to mimic the complex framework of native articular cartilage [50]. Hydrogel-based scaffolds offer an ideal platform for chondrogenesis due to their hydrophilic nature and ability to provide a stable environment conducive to cellular regeneration. Their biomechanical properties provide an adequate buffer, akin to native hyaline cartilage [51], which promotes essential cellular processes such as differentiation, migration, diffusion, and implantation [52]. Moreover, hydrogels can be tailored to mimic specific tissue extracellular matrices by adjusting parameters such as viscoelasticity, mechanical strength, stiffness, and swelling capacity [53].

Despite significant advancements in tissue engineering, only a handful of hydrogel-based materials have been clinically tested, including type I collagen, hyaluronic acid, and albumin–hyaluronic acid hydrogels, and the long-term success and durability of these implants remain to be seen, as reported by Kwon H et al. [54]. In addition to the hydrogel scaffolds already in clinical use, various hydrogel scaffolds have continued to be developed and tested for treating chondral and osteochondral injury over the past decade. Our review focuses specifically on studies that have tested chondrocyte-laden hydrogels in preclinical models, which are likely to inform future clinical investigations (Table 2). The aim of this analysis is to evaluate the strengths and weaknesses of each hydrogel scaffold, providing insights to guide the development of improved translational approaches for cartilage regeneration.

### 6.1. Natural-Polymer-Based Hydrogels

Natural-polymer-based hydrogels offer promising potential for cartilage tissue engineering due to their favorable characteristics, such as biocompatibility, biodegradability, and encoded bioactive patterns [78]. This section delves into recent advancements in the development of hydrogels to enhance ACI. We focus on the materials that have been tested in animal models, including alginate, agarose, chitosan, hyaluronic acid, fibrin, collagen, gelatin, silk fibroin, and their derivatives. We briefly explain the structures and the mechanism of supporting chondrogenesis of each material and summarize the reparative outcomes of each material along with the experimental details to allow the direct comparison of their potential in enhancing ACI.

**Alginate:** Derived primarily from brown seaweed and bacteria, alginate is a polysaccharide composed of 1,4-B-D mannuronic acid (MA) and 1,4-a-L-guluronic acid (GA), whose polymer properties depend on the ratio of these monomers [79]. Alginate’s natural biocompatibility, solubility, porosity, and degradability make it an attractive candidate for cartilage repair, including its potential application in ACI [30]. Experimental studies have shown that while alginate alone can facilitate the initial stages of chondrogenesis, it is more effective when combined with primary chondrocytes. In rabbit models, implants with alginate and chondrocytes exhibited significant articular cartilage regeneration, characterized by new isogenic chondral groups and chondral matrix formation [57]. Histological analysis revealed that animals with chondrocyte-enhanced alginate had a collagen fiber arrangement more akin to native cartilage and higher type II collagen expression compared to those with alginate alone. However, alginate’s limitations include insufficient mechanical and biological stability, primarily due to the lack of RGD molecules necessary for cell adhesion [80], which may trigger immunological responses [81]. To mitigate chondrocyte dedifferentiation, further studies explored the use of Tanshinone IIA (TIIA) with alginate, demonstrating that alginate combined with TIIA led to near-normal chondrocyte morphology, subchondral bone reconstruction, and hyaline-like collagen formation, showcasing its enhanced potential in cartilage repair applications [56].

**Agarose:** Agarose, a polysaccharide composed of alternating sequences of 1,3-linked β-D-galactose and 1,4-linked 3,6-anhydro-α-L-galactose, has demonstrated promising reparative outcomes in chondrogenesis, particularly for cartilage repair. Its biocompatibility and ability to create a supportive microenvironment with optimal hydration, mechanical stability, and nutrient diffusion are crucial for preserving the chondrocyte phenotype [82]. This microenvironment facilitates the maintenance of chondrocyte morphology and gene expression, promoting the synthesis of essential extracellular matrix components. Experimental studies have shown that incorporating low concentrations of agarose into hydrogels enhances nutrient diffusion and matrix deposition [83], crucial factors for effective cartilage repair. Furthermore, agarose hydrogels can be tailored to exhibit specific mechanical properties and can incorporate therapeutic agents, making them versatile tools in regenerative medicine [84]. However, challenges such as inadequate mechanical strength, premature degradation, limited diffusion, and potential inflammatory responses need to be addressed to optimize their use in clinical settings. Recent research explored the use of pulsed electromagnetic fields (PEMFs) to enhance the growth and healing of tissue-engineered cartilage grafts using agarose hydrogels [55]. In vitro studies with passaged adult canine chondrocytes embedded in agarose scaffolds showed that PEMF stimulation, particularly with coils oriented parallel to the articular surface, significantly improved repair stiffness and increased glycosaminoglycan (GAG) deposition compared to control groups. These findings were further supported by preliminary in vivo studies in a preclinical adult canine model, where PEMF-treated constructs exhibited higher likelihoods of normal chondrocyte and proteoglycan histological scores, suggesting potential clinical applicability for accelerated cartilage repair.

**Chitosan:** Derived from crustacean exoskeletons, chitosan is a polycationic polysaccharide known for its biocompatibility and biodegradability [85]. Its susceptibility to lysozyme degradation facilitates integration into biological systems, while its structural resemblance to cartilage extracellular matrix glycosaminoglycans highlights its potential in tissue engineering [8]. Chitosan hydrogels have demonstrated potential in cartilage repair due to their biocompatibility, biodegradability, and ability to interact with chondrocytes, supporting chondrogenesis and extracellular matrix formation. For example, in a preclinical study using chitosan hydrogels seeded with rabbit chondrocytes to repair articular cartilage defects, complete cartilage regeneration was seen after 12 weeks. In particular, the tissue showed close integration with the subchondral bone and exhibited morphological similarities to normal cartilage, and the International Cartilage Repair Society (ICRS) grading indicated better reparative outcomes for the tissue-engineered cartilage compared to control groups [58]. In addition, Herani-Tabasi et al. investigated injectable chitosan–hyaluronic acid (CS-HA) hydrogels combined with chondrocyte extracellular vesicles (EVs) [59]. EV treatment within the hydrogel scaffold increased the expression of key chondrogenic markers, such as SOX9 and COL2A1. It supported cartilage regeneration in vivo, showing that EV-treated chondrocytes in the CS-HA hydrogel had improved outcomes compared to untreated cells. Additionally, Chen et al. introduced a photocrosslinkable chitosan methacrylate (CHMA) and polyvinyl alcohol (PVA) hydrogel, which facilitated chondrocyte spheroid formation and maintained high cell viability [60]. In vivo results demonstrated cartilage regeneration in cylindrical defects in rabbits, with the hydrogel degrading within five weeks, suggesting its potential for clinical applications. While these findings indicate the usefulness of cartilage repair, certain challenges remain, including variable degradation rates, limited mechanical strength, and potential immunogenic responses, which require further investigation.

**Hyaluronic Acid (HA):** Hyaluronic acid (HA) hydrogels, derived from naturally occurring glycosaminoglycans [86], have been explored for cartilage repair due to their role in organizing the extracellular matrix, promoting chondrocyte growth, and enhancing tissue hydration [87]. However, HA’s rapid degradation and limited mechanical strength necessitate modifications, such as crosslinking or combining it with other materials, to improve its stability. In a study by Niemietz et al., human articular chondrocytes (HACs) were transplanted into full-thickness cartilage defects in minipigs using matrix-assisted HA hydrogels. Although HACs underwent chondrogenic redifferentiation in vivo, they failed to engraft in the porcine articular cartilage, with fewer than 5% of HACs persisting in the defect areas after two weeks. Despite this, early defect regeneration was observed, involving host cell invasion and the presence of collagen type II-rich tissue [69]. Another study by Hua et al. attempted to tackle the issue of HA’s mechanical limitations by developing a hybrid photocrosslinkable (HPC) hydrogel to withstand the hydraulic pressure of arthroscopic irrigation and to bind strongly to surrounding tissue. In vivo experiments on swine models showed that the HPC hydrogel could serve as a scaffold for arthroscopic autologous chondrocyte implantation, promoting long-term cartilage regeneration and integration. This system demonstrated mechanical strength sufficient for weight-bearing areas, making it a promising candidate for arthroscopic cartilage repair, where rapid gelation and tissue adhesion are crucial [70]. Another study by Zhu Y et al. introduced a photo-annealed granular hydrogel (GH) composed of hyaluronic acid, polyethylene glycol, and gelatin, specifically formulated to promote chondrocytes’ volume expansion and maintain their chondrogenic phenotype. In vitro, results demonstrated that GH improved chondrocyte morphology and increased matrix deposition compared to nongranular hydrogels (nGH). In vivo, studies in a rat full-thickness cartilage defect model showed that GH significantly stimulated hyaline-like cartilage regeneration, with enhanced matrix deposition and better integration into surrounding tissue. Mechanistic studies revealed that GH may activate the AMP-activated protein kinase/glycolysis axis, contributing to the improved chondrogenic phenotype of the encapsulated chondrocytes [71]. Overall, these studies illustrate HA hydrogels’ capacity to support chondrogenesis and cartilage regeneration, but challenges such as immune rejection and mechanical limitations still need to be addressed.

**Collagen:** Collagen hydrogels have emerged as a promising tool for cartilage regeneration, leveraging collagen’s natural role in maintaining the structure and function of articular cartilage, where collagen type II constitutes 90% of the cartilage’s dry weight [16]. Collagen interacts with chondrocytes through integrins, which initiate signaling cascades essential for chondrogenesis [88], the differentiation of precursor cells into chondrocytes, and extracellular matrix (ECM) formation [89]. Jiang et al. explored collagen–chondroitin sulfate hydrogels (CCHs) seeded with allogeneic chondrocytes to form ectopic cartilage tissue in a diffusion chamber. When transplanted into in vivo rabbit models, the CCH demonstrated improved cell proliferation, glycosaminoglycan (GAG) secretion, and expression of chondrocyte-specific markers like COL 2a1 and ACAN compared to pure collagen hydrogels. Histological evaluation revealed that CCHs more closely mimicked hyaline cartilage, with greater mechanical strength and enhanced integration into host tissue [61]. Mi Hyun Lim et al. further explored the use of human nasal chondrocytes (hNCs) embedded in collagen scaffolds for articular surface defect repair. hNCs have shown a high capacity for chondrogenesis in vitro, and in combination with collagen, they exhibited strong expression of cartilage-specific markers such as Aggrecan and SOX9. In an in vivo rodent model of osteochondral defects, hNC-collagen scaffolds facilitated substantial ECM synthesis, including proteoglycan-rich material, and supported the development of articular-like surfaces. Histological analysis showed that the implanted hNCs remained viable for up to eight weeks, contributing to long-term cartilage regeneration [62]. Further research by Jie Xie et al. focused on a hybrid hydrogel composed of collagen, hydroxyapatite (HA), and polyvinyl alcohol (PVA). The addition of collagen improved the biocompatibility and structural properties of the hydrogel, while the hydroxyapatite provided mechanical support. In vitro studies showed excellent cell viability and enhanced ECM formation when chondrocytes were seeded within the hydrogel. In vivo experiments in goat models revealed that COL-HA-PVA hydrogels effectively repaired osteochondral defects, particularly when pre-inoculated with chondrocytes [63]. Another innovative approach utilized collagen hydrogel microspheres containing allogeneic chondrocytes to fabricate artificial cartilage particulates (ACPs) for osteochondral repair in rabbits. The microsphere culture technique enabled the creation of cartilage constructs that could be implanted into osteochondral defects. The results indicated superior cartilage regeneration, improved integration into host tissue, and the recovery of subchondral bone volume. This method shows promise for future clinical applications, where microsphere-based collagen hydrogels could address the limitations of current tissue-engineered cartilage, such as poor mechanical properties [90] and tissue homogeneity [64].

**Gelatin:** Gelatin hydrogels, derived from collagen, are promising for cartilage repair due to their cell adhesion properties, which are driven by arginine–glycine–aspartic acid (RGD) sequences and matrix metalloproteinase (MMP) target sites that facilitate cell remodeling [91]. However, their poor mechanical properties and low thermal stability limit their effectiveness in cartilage regeneration [92]. To address these limitations, methacrylate gelatin (GelMA) was developed, which can be crosslinked via photopolymerization, offering biodegradability, biocompatibility, and tunability [93]. Despite these advances, GelMA hydrogels still fall short in achieving the high mechanical strength necessary for effective cartilage repair [94]. Experimental studies including one by Li-Shan Wang et al. have explored the role of stiffness in gelatin hydrogels. In one system, gelatin–hydroxyphenyl propionic acid (Gtn-HPA) hydrogels were developed with tunable stiffness ranging from 570 Pa to 2750 Pa. Chondrocytes encapsulated in these hydrogels demonstrated stiffness-dependent cellular functions. Specifically, chondrocytes in hydrogels with a stiffness of 1000 Pa exhibited the highest production of sulfated glycosaminoglycans (sGAG) and the most favorable gene expression ratios for type II to type I collagen, leading to enhanced cartilage tissue formation. In in vivo rabbit models of osteochondral defect repair, these medium-stiffness hydrogels promoted significant hyaline cartilage formation and better integration with surrounding tissue [67]. Another study by Chen-Chie Wang et al. introduced an expandable scaffold, utilizing microfluidic technology to enhance the integration of chondrocytes and scaffolds with recipient tissues. Gelatin-based scaffolds seeded with rabbit chondrocytes were implanted into osteochondral defects and assessed over six months. The chondrocyte/scaffold constructs showed substantial cartilage regeneration and superior integration with host tissue compared to untreated defects or autologous chondrocyte implantation. Biomechanical tests revealed that the scaffold-transplanted tissue had high compressive strength, approaching that of normal cartilage after six months. These expandable scaffolds achieved an 87% integration with host tissue, demonstrating their potential in enhancing chondrocyte integration and promoting cartilage repair [68]. However, GelMA hydrogels still lack the high mechanical properties required for efficient cartilage regeneration [95].

**Fibrin:** Fibrin, a polymer of fibrinogen molecules composed of α, β, and γ polypeptide chains joined by disulfide bridges in the N-terminal E domain, has long been utilized in surgery for its sealant and adhesive properties in natural wound healing [96]. The thrombin-mediated cleavage of the N-terminal fibrinopeptide, found in the fibrinogen α chain, initiates the assembly of polypeptides into fibrin. Given the crucial roles of fibrin and fibrinogen in blood clotting, the inflammatory response, and cell–matrix interactions, they do not induce toxic degradation or inflammatory responses [97]. Fibrin forms gels through the enzymatic polymerization of fibrinogen at room temperature in the presence of thrombin, with their degradation rate controllable by aprotinin, a proteinase inhibitor [98]. In the realm of cartilage regeneration, fibrin exhibits inherent biocompatibility and facilitates cellular adhesion, proliferation, and migration within hydrogel scaffolds [99]. Enriched with growth factors and cytokines, fibrin’s bioactivity further enhances cartilage repair processes such as chondrogenesis and angiogenesis [100]. Recent experimental approaches highlight the potential of fibrin-based constructs in cartilage tissue engineering. For instance, in a study investigating the repair of full-thickness cartilage defects in the femoral condyles of cynomolgus monkeys, a novel self-assembling peptide scaffold (IEIK13) was combined with articular chondrocytes treated with a chondrogenic cocktail (BMP-2, insulin, and T3, designated BIT) [65]. The results demonstrated that IEIK13 scaffolds, loaded with or devoid of chondrocytes, effectively supported cartilage regeneration. In vitro studies revealed that IEIK13 supports the production of cartilage by BIT-treated chondrocytes, while in vivo results showed successful integration of the implant, as confirmed by histological analysis and immunohistochemical staining three months post-implantation. Another promising approach involves the stratified zonal implantation of autologous chondrocytes for improved cartilage repair. In an in vitro porcine model of chondral defects, chondrocytes isolated from different zones of articular cartilage were expanded using dynamic microcarrier culture (dMC) and then separated by a spiral microchannel sorting method. These zonal chondrocytes were implanted as a bilayer fibrin hydrogel construct in the knee. Six months after implantation, the bilayer approach not only improved cartilage repair outcomes but also recapitulated the zonal architecture, including chondrocyte arrangement, proteoglycan 4 distribution, and collagen alignment [66]. These findings suggest that zonal chondrocyte implantation within fibrin hydrogels can enhance the functional and structural integration of repaired cartilage, offering significant advancements in ACI techniques. However, limits arise from fibrin’s rapid degradation, variable mechanical properties, and the potential risk of fibrosis associated with prolonged exposure, which may compromise the long-term stability and efficacy of cartilage repair [101]. Strategies such as crosslinking fibrin hydrogels or incorporating reinforcing agents are being explored to address these limitations and enhance the durability of fibrin-based constructs for cartilage tissue engineering [102].

**Silk Fibroin:** Silk fibroin is a natural polymeric biomolecule composed of two proteins hydrophobic fibroin and hydrophilic sericin. One attribute that makes it attractive for tissue engineering is that it is a sustainable and natural source as it is derived from silkworms [103]. This natural material possesses excellent biocompatibility, with less toxic degradation products, and an absence of adverse immune responses within the host system. Many studies have shown that silk fibroin maintains the morphology of chondrocytes and enhances cellular viability [103]. Furthermore, its tunable mechanical properties enable the customization of scaffolds to match the mechanical requirements of native cartilage [104]. In addition, its excellent biocompatibility and slow degradability allow for prolonged structural support within the defect site, facilitating the gradual regeneration of cartilage tissue [105]. Nevertheless, challenges remain in optimizing hydrogel formulation to balance degradation rates, addressing poor swelling properties, and enhancing the ability to attach and proliferate certain cells. To address these limitations, studies have used n-butanol as a self-emulsifier to create 3D porous scaffolds to adjust pore sizes. In the study, researchers indicated that RSF-based scaffolds (14 wt%) possessed sufficient viscosity and biomechanical stability to fill gaps and to ensure tight integration to allow for improved lateral integration between the host tissue and the implanted scaffold. In vivo results in osteochondral models using rabbits demonstrated increased production of type II collagen, as well as increased cell migration and enhanced chondrocyte activity. Furthermore, the hydrogel demonstrated enhanced mechanical strength and flexibility due to the β-sheet transformation induced by physical forces (compression, tensile, shear) indicating its ability to serve as a medium for nutrient exchange and signal transmission aiding in the sustaining of chondrocytes post-implantation [75].

### 6.2. Synthetic-Polymer-Based Hydrogels

While hydrogels based on naturally derived polymers show excellent biocompatibility for chondrocyte growth, proliferation, and phenotype maintenance, the low mechanical properties and uncontrolled degradation often limit their applications. Hydrogels based on synthetic polymers, on the other hand, exhibit excellent characteristics in terms of molecular weight, degradation, and mechanical properties [106]. This section delves into recent advancements in the development of hydrogels using synthetic biomaterials, emphasizing their application in vivo for the repair of osteochondral defects in animal models.

**Polyethylene glycol (PEG):** In the structure of polyethylene glycol (PEG), the key feature that allows for precise control over hydrogel properties is its highly tunable and versatile chemical structure. PEG is a linear, synthetic polymer composed of repeating units of ethylene glycol (-CH2-CH2-O-), which gives it a flexible and hydrophilic backbone [107]. The molecular weight of PEG can vary widely, providing control over the size and mesh density of the resulting hydrogel network [108]. Chemical modification of PEG can further enhance its properties and functionalities. For example, PEG can be functionalized with various chemical groups, such as methacrylate or acrylate, to enable crosslinking through photopolymerization or other chemistries [109]. This allows researchers to precisely control the degree of crosslinking and thus modulate the mechanical strength and stability of the hydrogel. Moreover, PEG can be conjugated with bioactive molecules, such as cell-adhesive peptides or growth factors, to promote specific cellular responses within the hydrogel matrix [110]. These bioactive modifications enable control over cell adhesion, proliferation, and differentiation within the hydrogel, enhancing its suitability for tissue engineering applications. For example, experimental results from Wang et al. using PEG hydrogels functionalized with vinyl sulfone and short dithiol crosslinkers showed a local maximum compressive strength of ∼20 MPa when gelation occurred at the overlap concentration. This provided a mechanically strong yet light scaffold. In addition, chondrocyte-laden PEG hydrogels, when transplanted into an in vivo osteochondral defect model in SCID mice, promoted chondrocyte proliferation and ECM production, resulting in newly formed hyaline cartilage that integrated with the host tissue [74]. However, PEG-based hydrogels have limitations due to their biologically inert nature [111]. Unlike natural ECM components, PEG lacks intrinsic cell-adhesive motifs, hindering cell adhesion, proliferation, and differentiation necessary for tissue regeneration. In addition, PEG’s nondegradable nature raises concerns about prolonged scaffold resistance in the body, potentially interfering with tissue remodeling and integration.

**Polyvinyl Alcohol (PVA):** PVA, derived from the hydrolysis of polyvinyl acetates, has garnered attention due to its notable characteristics, including, high swelling capacity, porosity, and viscoelasticity [112]. PVA-based hydrogels offer tunable mechanical properties, chemical stability, adhesive capability, and straightforward preparation methods, contributing to their versatility in cartilage regeneration applications [113]. The high swelling capacity of PVA hydrogels facilitates water absorption and retention within the matrix, while their porous structure enhances diffusion and supports tissue ingrowth. Furthermore, PVA hydrogels exhibit viscoelastic behavior, providing mechanical support to the surrounding tissues and allowing for the adjustment of mechanical properties to match those of native cartilage [114]. In addition, they demonstrate excellent chemical stability over time and possess adhesive capabilities, ensuring stable integration with surrounding tissue [115]. Moreover, their simple preparation methods, such as solution casting or crosslinking techniques, make them suitable for large-scale manufacturing and clinical translation [116]. However, as seen with other synthetic polymers, limitations in biocompatibility and microstructure pose challenges in supporting cellular activities for cartilage regeneration.

**Poly(ε-caprolactone) (PCL):** Poly(ε-caprolactone) (PCL), a semi-crystalline synthetic polyester, exhibits a slow degradation rate, rendering it ideal for long-term structural support in tissue engineering applications [117]. Furthermore, its mechanical strength and flexibility closely resemble those of native cartilage, making it suitable for load-bearing environments and supporting chondrogenic differentiation [118]. However, challenges arise from PCL’s hydrophobic nature, which can hinder cell adhesion and migration within hydrogel matrices. Experiments have explored the efficacy of bi-phasic, fiber-reinforced PCL scaffolds for regenerating both articular cartilage and subchondral bone. For instance, fiber-reinforced cartilaginous constructs, combining PCL with alginate hydrogels have demonstrated the ability to support chondrogenesis in vitro and promote endochondral bone formation in vivo [76]. These bi-phasic templates were evaluated in a large animal model, revealing that PCL-based scaffolds led to the development of phenotypically stable cartilage over vascularized endochondral bone. However, the quality of repair varied, suggesting the need for further refinement of the PCL constructs to improve outcome consistency. Moreover, the zonal organization of cartilage, which is critical for functional and biomechanical integrity, poses an additional challenge for PCL-based scaffolds. A study investigating zonal approaches with PCL constructs in a minipig model highlighted the importance of layering strategies to mimic the native cartilage structure [73]. While PCL-based scaffolds supported initial chondrogenesis, long-term outcomes revealed bone erosion and misplacement of grafts during joint loading. This issue suggests that although PCL’s mechanical strength is beneficial for load-bearing applications, its enforcement within the subchondral bone may contribute to complications in vivo. Despite these challenges, PCL’s slow degradation rate and mechanical properties continue to make it an attractive candidate for cartilage repair, particularly in environments requiring long-term stability. Ongoing research is focusing on optimizing surface modifications and hybrid strategies (such as blending with hydrophilic polymers) to address its hydrophobicity and improve cellular integration for enhanced cartilage regeneration [119].

**Polylactic Acid (PLA):** Poly (lactic acid) (PLA), a biodegradable polyester derived from renewable resources such as corn starch or sugarcane, has garnered interest in cartilage regeneration due to its excellent biocompatibility and controlled degradation kinetics [120]. PLA hydrogels can be precisely engineered to provide a controlled release of bioactive molecules, facilitating tissue healing and regeneration [121]. However, PLA’s inherent brittleness and the acidic degradation byproducts generated during its breakdown pose challenges for cartilage repair [122]. The brittleness of PLA may limit its utility in load-bearing applications, where mechanical strength is crucial for long-term durability [123]. Additionally, the acidic degradation byproducts may induce inflammation and impair the microenvironment necessary for cartilage regeneration. To address these limitations, strategies are being explored to enhance the mechanical properties of PLA-based hydrogels, such as blending with other polymers or incorporating reinforcing agents [124]. Furthermore, methods to mitigate acidic degradation, such as adjusting the PLA composition or incorporating alkaline modifiers, are being investigated to prolong the effectiveness of PLA hydrogels in cartilage repair.

**Poly (lactic-co-glycolic acid) (PLGA):** Poly (lactic-co-glycolic acid) (PLGA), a copolymer of lactic acid and glycolic acid, offers a combination of the advantages of PLA and PGA, providing tunable degradation rates and mechanical properties [125]. Depending on the ratio of lactide to glycoside acid used for the polymerization, different forms of PLGA can be obtained, allowing for tunable physiochemical properties. Like PLA and PGA, the chemical properties of PLGA allow hydrolytic degradation through de-esterification; once degraded, the monomer components of each polymer are removed by natural pathways. PLGA hydrogels have demonstrated promise in cartilage tissue engineering owing to their biodegradability, biocompatibility, and capability to deliver therapeutic agents [126]. In one study, PLGA, along with PCL and PLA, was used to reinforce alginate hydrogels and support mesenchymal stem cell (MSC) chondrogenesis. These reinforced hydrogels formed the endochondral bone phase of bi-phasic osteochondral constructs, which also included a cartilage layer engineered from infrapatellar fat pad stem cells (FPSCs) and chondrocytes. Following implantation in animal models, the constructs supported vascularized endochondral bone formation and stable cartilage [76]. Furthermore, in vivo bi-phasic constructs led to more hyaline-like cartilage repair after six months compared to commercial scaffolds, though the quality of repair varied, highlighting the need for further optimization of implant designs. However, challenges such as the generation of acidic degradation byproducts and rapid initial degradation kinetics may affect their suitability for long-term cartilage repair, particularly in load-bearing joints where prolonged mechanical stability is crucial.

Autologous chondrocyte implantation (ACI) has explored various biomaterials, each offering unique advantages and facing specific challenges. Alginate, agarose, chitosan, hyaluronic acid, collagen, gelatin, fibrin, and silk fibroin all demonstrate notable biocompatibility and support for chondrogenesis. Alginate’s natural biocompatibility and porosity facilitate initial chondrogenesis; however, it is limited by insufficient mechanical stability and cell adhesion. Agarose, while providing a supportive microenvironment and promoting extracellular matrix (ECM) formation, struggles with mechanical strength and potential inflammatory responses. Chitosan’s resemblance to cartilage ECM supports chondrocyte interactions, although challenges with degradation rates and mechanical limitations persist. Hyaluronic acid enhances tissue hydration and chondrocyte growth, but its rapid degradation requires crosslinking to improve mechanical stability. Collagen hydrogels effectively mimic cartilage structure, promoting ECM formation; however, they also need mechanical reinforcement to ensure long-term durability. Gelatin, derived from collagen, benefits from cell adhesion properties but is limited by poor mechanical properties and thermal stability. Fibrin facilitates cellular adhesion and cartilage repair but degrades rapidly and may induce fibrosis. Lastly, silk fibroin’s tunable mechanical properties and biocompatibility make it an attractive option, although balancing its degradation rate and improving cell attachment remain key challenges.

Synthetic materials such as PEG, PVA, PCL, PLA, and PLGA have unique advantages and limitations in cartilage repair applications. PEG offers a highly tunable and versatile chemical structure, allowing precise control over hydrogel properties such as mechanical strength and stability. However, PEG’s biologically inert nature limits cell adhesion, proliferation, and differentiation, and its nondegradable nature raises concerns about long-term scaffold resistance. PVA-based hydrogels, known for their tunable mechanical properties and chemical stability, facilitate water retention, diffusion, and tissue ingrowth. Despite their straightforward preparation and good integration with surrounding tissues, PVA’s biocompatibility and microstructural challenges still pose limitations for cellular activities in cartilage regeneration. Poly(ε-caprolactone) (PCL) scaffolds, particularly those that are bi-phasic and fiber-reinforced, support chondrogenesis and promote endochondral bone formation in large animal models. Nevertheless, issues such as the zonal organization of cartilage and potential bone erosion during joint loading highlight the need for further refinement. PLA and its copolymer, PLGA, are of interest for their biocompatibility and controlled degradation kinetics. PLA’s controlled release of bioactive molecules facilitates tissue healing but is hindered by brittleness and acidic degradation byproducts. PLGA, with its tunable degradation rates and mechanical properties, has shown promise, especially when combined with materials like PCL and alginate. While PLGA-reinforced constructs have demonstrated potential in forming hyaline-like cartilage, challenges such as acidic byproducts and rapid initial degradation kinetics emphasize the need for ongoing optimization to improve long-term efficacy in cartilage repair.

Each biomaterial holds promise for ACI surgery, but further optimization is needed to address their limitations and enhance their reparative potential.

## 7. Fixation and Integration of Implants with Native Cartilage

**Figure 2:**
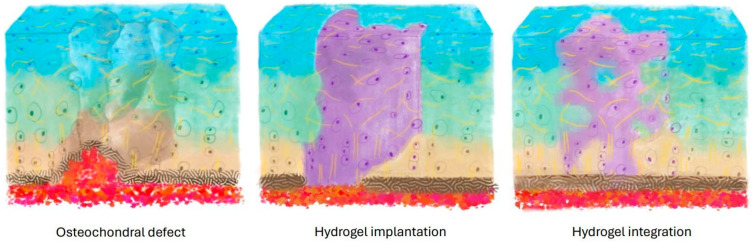
A successful ACI-based cartilage repair needs to have efficacious fixation and integration of hydrogels and implants.

Fixation and integration of hydrogels within cartilage defects are critical for several reasons (Figure 2). Firstly, they ensure mechanical stability, averting implant displacement that could trigger inflammation and hinder proper healing [127]. Secondly, seamless integration promotes the effective transfer of mechanical loads between the implanted construct and surrounding tissue, fostering functional restoration of the joint. Lastly, integration facilitates nutrient exchange and signaling molecule exchange, crucial for sustaining cell viability, proliferation, and differentiation within the defect site [22]. Nevertheless, achieving robust hydrogel-based integration within cartilage defects remains a significant challenge [128].

The knee joint, along with other weight-bearing joints, undergoes significant mechanical stress, compounded by the intricate geometry of cartilage surfaces, creating challenges for securing and reinforcing hydrogel-based constructs. Consequently, the initial effective fixation of hydrogels at defect sites becomes paramount for successful integration. Various fixation methods have been studied, including glue, BioGlue, and direct suturing of non-enforced and enforced constructs [129]. While press-fit is commonly employed, it carries the risk of chondrocyte apoptosis and necrosis, resulting in a hypocellular region around the defect [130]. Alternatively, bioresorbable pins offer a viable solution, demonstrating no adverse events in either the short or the long term [131]. However, recent research suggests that this method may induce more subchondral bone remodeling and inferior cartilage repair compared to fibrin glue [132].

Recent scientific literature has extensively addressed strategies to overcome the hurdle. For instance, the development of specialized ‘glues’ by functionalizing chondroitin sulfate with methacrylate and aldehyde groups has enabled the formation of chemical bonds with both native tissue and an acrylate-based scaffold. This innovative approach, as explored by researchers such as Schuurmans et al., enhances the stability and integration of the cartilage graft [133]. Moreover, in vivo animal model experiments conducted by Wang et al. showcased the successful integration of hydrogels with cartilage tissue in the presence of the chondroitin sulfate adhesive, resulting in enhanced tissue development and repair compared to controls [134]. Furthermore, mechanical testing demonstrated improved stability of the adhesive against tensile and shear forces. Similarly, research by Sharma et al. highlighted in vivo improvements in clot formation and mechanical strength with the chondroitin sulfate adhesive-hydrogel system, enhancing defect fill. Subsequent human cadaveric studies and a pilot clinical trial further supported the safety and efficacy of this approach, with magnetic resonance imaging confirming enhanced tissue integration and reduced pain in treated patients [135]. One factor contributing to the difficulty of the adhesive behavior of hydrogels, as mentioned by Zhang et al., is the large amount of water in their polymer network, creating a weak boundary layer that can prevent direct surface contact between the hydrogel and substrates, leading to diminished surface energy and deterioration of the adhesion strength. Therefore, inspired by mussel adhesion, a variety of catechol-functionalized adhesive hydrogels have been developed to overcome the unfavorable wet cartilage environment resulting in higher bonding strength [136]. Furthermore, spider aggregate glue [137] and tissue-bonding dry double-sided tape [138] have been used to improve the adhesion of hydrogels.

In addition to these methods, other innovative approaches have been reported. Kuang et al. infiltrated photocrosslinkable [Poly-d, l-lactic acid/polyethyleneglycol/poly-d, l-lactic acid] (PDLLA-PEG) and a photoinitiator into cartilage and then applied visible light onto the constructs. Interestingly, an interconnected and continuous hydrogel structure that fixes the implant within the host cartilage is formed [139]. Furthermore, Arvayo et al. showed promising enhanced integration of articular cartilage grafts via photochemical bonding which employs light-activated photosensitizers to excite and covalently crosslink proteins across the implant–host interface followed by mild enzymatic digestion [140]. Given the collage nanofibers that anchor cartilage to the surface of the bone, Zhao et al. devised NEST (nanofiber-enhanced sticking) which takes a nanofibrous bacterial cellulose sheet bonded to a porous base with a hydroxyapatite-forming cement followed by infiltration of the nanofibrous sheet with hydrogel-forming polymeric materials. This approach creates a mineralized nanofiber bond that mimics the structure of the osteochondral junction, in which collagen nanofibers extend from cartilage into a mineralized region that anchors cartilage to bone showing improved bonding [141].

After implantation, hydrogel scaffolds face several challenges in achieving integration with native cartilage. Pre-digestion rinses including using reagents such as hyaluronidase, collagenase, and chondroitinase ABC have emerged as a promising tactic to break down the dense matrix at the border of grafts, facilitating cell migration to the host–graft junction and promoting integration. Research by Janssen et al. has shown the effectiveness of digestion rinses in enhancing graft integration and overall outcomes in cartilage repair procedures [142]. In addition, because of the low cell density surrounding defects, methods to actively recruit cells into the edge of the graft have been utilized including the use of chemotactic agents such as platelet growth factor (PDGF), insulin-like growth factor I (IGF-1), basic fibroblast growth factor (bFGF), vascular endothelial growth factor (VEGF), and bone morphogenic proteins (BMPs). Strategies to enhance later integration include anti-apoptosis agents, including a study by Gilbert et al. which showed that short-term intra-articular administration of potent caspase inhibitor (Z-VAD-FMK, ZVF) results in a decrease in chondrocyte apoptosis in vitro leading to a decrease in cartilage degeneration [143].

Another approach for promoting integration involves inducing collagen crosslinking across cartilage interfaces using the enzyme lysyl oxidase (LOX). The stabilization of collagen fibrils relies on the formation of collagen crosslinks. In articular cartilage, key collagen crosslinks associated with growth and maturation include the difunctional crosslink, dehydrodihydroxylysinonorleucine, and the trifunctional crosslink, hydroxylysyl pyridinoline. The metabolic pathway responsible for the formation of these crosslinks is contingent upon the enzyme lysyl oxidase, which catalyzes the conversion of lysine and hydroxylysine amines into their aldehyde forms, subsequently enabling them to react and form crosslinks. Ahsan et al. were among the first to demonstrate the significance of lysyl oxidase-mediated crosslinks in resisting collagen extraction and promoting integrative cartilage repair, utilizing cartilage explants from cows treated with b-aminopropionitrile [144]. Subsequently, Athens et al. investigated the potential of LOX to alter integration in both native-to-construct and native-to-native tissue systems [145]. Additionally, treatment with ribose, which induces advanced glycation end-product crosslinks between collagen fibers, has shown promise in altering the tensile, compressive, and shear moduli of articular cartilage. Moreover, Hunter et al. conducted a comparative study on four different engineered cartilages in a hybrid culture system. Interestingly, they found that the interfacial strength of tissue-engineered cartilages did not correlate to measures of gross biochemical content (collagen, proteoglycan, or cell content), suggesting the need for alternative predictors [146].

Implants encounter challenges not only in integrating with surrounding native cartilage tissues but also in anchoring effectively to the underlying bone tissue. While hydrogels serve as supportive matrices for cartilage regeneration, establishing proper anchorage to subchondral bone is crucial for ensuring long-term joint stability. Additionally, maintaining the integrity of the interface between cartilage and bone is essential for preserving mechanical function. Despite their distinct mechanical properties, this interface must withstand loading to prevent stress concentration, delamination, or failure of the repair tissue. Internal geometry significantly influences the design of hydrogel scaffolds for cartilage–bone integration. Carefully engineered pore architectures are necessary to facilitate cell infiltration and the exchange of nutrients and waste products within the tissue-engineered construct [9]. Furthermore, scaffold pore sizes must be tailored to accommodate the unique vascularization requirements of both cartilage and bone tissues. Studies have underscored the importance of scaffold pore size and distribution in regulating cell behavior and tissue formation [147]. Smaller pore sizes promote cell adhesion and proliferation, while larger pores facilitate nutrient diffusion and waste removal [148]. Hence, striking a balance is crucial to ensure adequate cell infiltration and tissue regeneration throughout the scaffold.

To address these challenges, researchers have explored various strategies to optimize scaffold geometry and pore architecture. Surface modifications, such as incorporating bioactive molecules or nano-topographical cues, can enhance cell adhesion and migration within the scaffold [149]. Pore architecture drives cell migration throughout the scaffold by providing ample nutrient and waste transport while simultaneously providing surface area for cell attachment and growth. Additionally, the attachment of cells is further enhanced by specific surface textures that aid the attachment of specific cells and help guide cell growth and differentiation. These modifications promote cellular interactions with the scaffold material, facilitating tissue regeneration and integration with native cartilage and bone tissues. In addition to surface modifications, the incorporation of growth factors has shown promise in stimulating tissue regeneration and promoting cartilage–bone integration. Growth factors such as transforming growth factor-beta (TGF-β) and bone morphogenetic proteins (BMPs) play key roles in chondrogenesis and osteogenesis, respectively [150]. By incorporating these growth factors into the scaffold, researchers can create a microenvironment conducive to tissue regeneration and cartilage–bone integration. Furthermore, engineering scaffolds with gradient properties allow for the creation of biomimetic environments that mimic the native tissue architecture. Gradient scaffolds can replicate the transition from cartilage to bone physicochemical properties and stress transmissions, providing cues for cell differentiation and tissue formation which in turn affect the viscoelasticity and stiffness of the extracellular matrix [151]. These scaffolds aid in promoting integration between cartilage and bone tissues, enhancing the overall functionality and stability of the tissue-engineered construct. To address inflammation-related challenges, targeted strategies, such as the use of single-chain variable fragments (scFVs) against ROS-modified collagen type II, have been explored to selectively target and repair damaged cartilage regions [152].

## 8. Conclusions

Autologous-assisted chondrocyte implantation (ACI) and matrix-assisted autologous chondrocyte implantation (MACI) present a promising approach for treating cartilage injury across various joints, including the knee, hip, shoulder, and ankle. However, significant challenges remain. A primary concern is the current limitation of hydrogels in mimicking the viscoelastic properties of native cartilage, which is crucial for diverse joint applications. Many hydrogels fail to replicate the zonal architecture and complex collagen network inherent in native cartilage, affecting their ability to endure repetitive loading in different joint environments [153]. This discrepancy can lead to inadequate load-bearing capacity and potential delamination at the interface between the hydrogel and native tissue, a risk particularly pertinent to weight-bearing joints.

Achieving mechanical properties that match those of native tissue while ensuring biocompatibility remains the crux of cartilage tissue engineering across joints. Effective integration of hydrogels with native cartilage is crucial for restoring joint function, preventing further degeneration, and promoting long-term durability of the repair [128]. However, robust integration remains a primary barrier to the clinical translation of hydrogels in ACI and MACI. Successful integration with native cartilage relies heavily on hydrogel’s ability to facilitate cell infiltration and tissue regeneration, critical for joints subjected to high mobility or load.

Challenges arise in promoting chondrocyte migration and proliferation throughout the scaffold [88], as poor cell distribution can result in uneven tissue formation and compromised mechanical properties. One promising strategy is the use of chemotactic agents, which can attract viable cells into the interface between the graft and native tissue, thereby enhancing cell migration and integration. Studies have demonstrated the efficacy of chemotactic agents in improving graft–host integration, making them a valuable tool in cartilage repair [154]. Addressing these challenges demands innovative approaches to enhance integration, such as scaffold design optimization, surface modifications, and incorporation of growth factors. Moreover, comprehensive preclinical testing, including rigorous mechanical characterization and evaluation of integration mechanisms, is essential to assess the performance of hydrogel scaffolds in relevant animal models. By overcoming the barrier of integration, hydrogel-based scaffolds hold tremendous promise for advancing ACI techniques and offer viable solutions for cartilage repair in multiple joint applications in clinical settings.

## Figures and Tables

**Table 1:** ICRS grading system to evaluate cartilage injury.

Grade 1	Soft indentations and/or superficial cracks
Grade 2	Small cracks or lesions that extend to less than half of the cartilage depth
Grade 3	Deep cracks or gaps exceeding 50% of the cartilage depth
Grade 4	Cracks extending the entire thickness of the cartilage to the underlying bone
Grade 5	Defects encompassing full thickness of cartilage, involving the subchondral bone

**Table 2:** Animal studies assessing chondrocyte-laden hydrogels for treating chondral/osteochondral injury (from 2014 to 2024). * Citations: 1 [55], 2 [56], 3 [57], 4 [58], 5 [59], 6 [60], 7 [61], 8 [62], 9 [63], 10 [64], 11 [65], 12 [66], 13 [67], 14 [68], 15 [69], 16 [70], 17 [71], 18 [72], 19 [73], 20 [74], 21 [75], 22 [76], 23 [77].

Material	Cell Source	Animal Model	Defect Size (⌀ × Depth)	Seeding Density (Cells/mL)	Weeks	Summary + Outcome Measures (ICRS/OARSI/Young’s Modulus)		* Ref.
Agarose	dogs	dogs	6 mm × 1 mm	30 × 10^6^	13	Application of pulsed electromagnetic fields to tissue-engineered scaffolds resulted in increased outcome scores and enhanced repair outcomes, irrespective of the implantation or microfracture procedures utilized.	**OARSI** Scores at 13 weeks: TE + PEMF = 19, TE-PEMF = 18.5 **KEY: TE** = tissue engineered scaffolds, PEMF = pulsed electromagnetic fields, **+/−** = with/without **PEMF**	1
Alginate, sodium hyaluronate (Alg-HA)	rats	rats	0.8 mm × 1.2–1.5 mm	4 × 10^5^	3	The administration of TIIA through scaffolds in pharmacological applications demonstrated protective effects against chondrocyte dedifferentiation. Furthermore, they pinpointed several regulatory pathways, with SOX6 emerging as a primary candidate believed to directly regulate the chondrocyte dedifferentiation process.	N/A—histology and immunohistochemistry	2
Alginate	rabbits	rabbits	3 mm × 1 mm	2 × 10^6^	13	In contrast to blank scaffolds and primary chondrocytes, hDPSCs exhibited superior chondrogenic outcomes. These results could be attributed to the potential anti-inflammatory properties of hDPSCs, as well as the duration of incubation in a chondrogenic medium.	N/A—histology and immunohistochemistry	3
Chitosan	rabbits	rabbits	4 mm × 3 mm	1 × 10^7^	4, 8, 12	The experimental group exhibited notably higher average values in both gross and histological assessments (*p* < 0.05). The authors observed that repair occurred independently with pure chitosan hydrogels.	**ICRS** Morphology Scores at 12 weeks: Control = ~6, Blank = 7, Experimental = ~10 Histology Scores at 12 weeks: Control = 10, Blank = 5, Experimental = 16	4
Chitosan, hyaluronic acid (CS-HA)	TKA human patients	rabbits	4 mm × 3 mm	7 × 10^5^	4, 24	Among the various scaffolds examined, the CS-HA/EV/MSC group displayed moderately encouraging ICRS (MORPH SCORE = 2.2) after 4 weeks. However, after 24 weeks, it demonstrated scores most akin to “normal” unimpaired cartilage (MORPH SCORE = 4) in comparison to other experimental scaffold combinations.	**ICRS** Gross Morphology Scores at 24 weeks via MRI evaluation (*p* < 0.01 and *p*< 0.0001): EV/MSC = 2.65, MSC = 1.85, CS-HA = 0.65, Control = 0.55 Histology Scores at 24 weeks: Normal = 3, Control = 0, CS-HA = 0, MSC = 1.1, EV/MSC = 2.15, CS-HA/MSC = 2.9, CS-HA/EV/MSC = 3	5
Chitosan methacrylate, PVA (CHMA-PVA)	rabbits	rabbits	4 mm × 4 mm	1 × 10^7^	8	Initial findings indicated cartilage regeneration when compared to the control group. The CHMA-PVA hydrogels exhibited promising features including complete closure, effective integration of superficial neocartilage, and manageable inflammation, suggesting their potential as biocompatible scaffolds for accelerated regeneration.	N/A—histology and immunohistochemistry, rheological testing	6
Chondroitin sulfate/collagen/hyaluronic acid (CCH)	rabbits	rabbits	3 mm × 2 mm	5 × 10^7^	4, 8, 12	Consistently, the CCH scaffold maintained significantly higher scores compared to both the collagen group and the control group (*p* < 0.05). Notably, while collagen showed clear margins, the edges of the CCH group were indistinguishable at multiple points.	**ICRS** Gross Morphology Scores at 12 weeks: Control = 11, CCH = 13, C = ~11 **KEY:** **C** = cell-collagen group, **CCH** = cell-collagen chondroitin sulfate/HA group (conducted at 1, 2, and 3 months by blinded scorers)	7
Collagen	human patient donors	rats	2 mm × 2 mm	1 × 10^6^	8	In animal defect models, hNC collagen hydrogels demonstrated enhanced proteoglycan production and glycosaminoglycan (GAG) synthesis compared to the collagen control group.	N/A—histology and immunohistofluorescence	8
Collagen, hydroxyapatite, PVA (COL-HA-PVA)	goats	goats	8 mm × 8 mm	1 × 10^6^	4	The COL-HA-PVA hydrogel exhibited good biocompatibility and integration compared to the cell-free hydrogel and the empty control. In addition, it was noted that the goat model may have some self-regenerative properties by exhibiting some regeneration in the empty control group based on histology results.	N/A—histology and spectrophotometry	9
Collagen hydrogel microspheres, artificial cartilage particulates (ACPs)	rabbits	rabbits	4 mm × 2.5 mm	1 × 10^6^	4, 13	Artificial cartilage particulates (ACPs) within collagen hydrogels demonstrated comparable repair outcomes to native tissue. Notably, there was significantly improved cell migration observed after 7 days compared to 14 days of in vitro culture. Acknowledging limitations, the authors aim to explore alternative materials to enhance outcomes.	**Young’s modulus** Collagen hydrogel = 22 kPa ACP 7 days = 81 kPa ACP 14 days = 115 kPa	10
Fibrin and IEIK13	monkeys	monkeys	3.5 mm	1 × 10^6^	12	The incorporation of IEIK13 into scaffolds that were empty or cell-laden with chondrocytes produced similar and comparable results. This outcome supports the incorporation of EIK13 in regenerative efforts of osteochondral defects.	**ICRS** Only the quality of repair was assessed in the three animal subjects. Model 1 and Model 2 defects were classified as grade II (nearly normal), while Model 3 was graded as grade III (abnormal repair). The outcomes comparing seeded versus acellular scaffolds were depicted through images rather than numerical scores.	11
Fibrin	mini pigs	mini pigs	3 mm × 1–2 mm 6 mm × 1 mm	1 × 10^7^	26	Encouraging outcomes regarding zonal chondrocyte architecture and the formation of healthy subchondral bone support the effectiveness of a stratified zonal chondrocyte implantation approach.	**Young’s modulus** Full thickness: WB = < 0.1 MPa, NWB = ~0.3 MPa Medium/large chondrocytes: WB = ~0.25 MPa, NWB = ~0.25 MPa	12
Gelatin-hydroxyphenyl propionic acid (Gtn-HPA)	rabbits	rabbits	2 mm × 1 mm	1 × 10^7^	4, 12	In comparing hydrogels with different stiffness levels, discovered that medium-stiffness hydrogels yielded favorable regenerative results in defect models compared to low- and high-stiffness ones. Additionally, the cellular processes of chondrocytes were found to be influenced by the stiffness of the hydrogel.	N/A—Wakitani, histological grading scale, rheological assessment	13
Gelatin, microbubbles	rabbits	rabbits	3 mm × 3 mm	1 × 10^6^	8, 17, 26	The expandable hydrogel model demonstrated encouraging outcomes, achieving an 87% integration at the interfaces over a period of up to 6 months. Moreover, the hydrogel effectively absorbed high compressive forces comparable to those experienced by normal cartilage in the defect models.	**Young’s modulus** at 8 weeks: S = 0.22 C + S = 0.41 Control = N/A at 17 weeks: S = 0.27 C + S = 0.59 Control = 0.89 at 26 weeks: S = 0.32 C + S = 0.83 Control = N/A **KEY:** S = scaffold only (blank), C + S = cells + scaffold	14
Hyaluronic acid–albumin Novocart Inject and fibrin glue	human patient donors	mini pigs	6 mm diameter	1 × 10^6^	2, 4	Hydrogels containing human articular cartilage experienced rapid degeneration within 2 weeks post-implantation, accompanied by the infiltration of surrounding macrophages. Consequently, these grafts were rejected in the animal models, leading to a lack of cartilage regeneration.	N/A—blinded histomorphometry evaluation and modified O’Driscoll scores	15
Hyaluronic acid, MES, and NB	pigs	pigs	7 mm diameter (full thickness)	100 × 10^6^	4, 13, 26	Following implantation, the hydrogel exhibited indications of successful cartilage regeneration; however, excessive, and hypertrophic cartilage growth was noted. It was therefore concluded that the hydrogel was biocompatible and viable, but the use of BMSCs was proposed instead of chondrocytes due to their dual osteogenic and chondrogenic characteristics.	**Young’s modulus** Normal = 22.5 MPa, Control = ~14 MPa, Experimental = ~23 MPa	16
Hyaluronic acid, PEG, and gelatin	rats	rats	2 mm × 2 mm	1 × 10^7^	6, 12	Granular hydrogel (GH) scaffolds replicated cartilage that closely resembled healthy cartilage, whereas nongranular (nGH) scaffolds exhibited a more fibrous appearance. At 12 weeks, GH achieved higher scores compared to nGH. These scores were further analyzed across categories, including matrix staining, cell morphology, surface architecture, basal integration, tissue morphology, and chondrocyte clustering.	**ICRS** Gross Morphology Scores at 12 weeks: GH = 8.6 nGH = 8.1 Histology Scores at 12 weeks (*p* < 0.05): Control = 24.2 GH = 65 nGH = 31.9	17
CSA-NH2, Odex, and HA-PNIPAAm	human patient donors	rabbits	3 mm × 2 mm	5 × 10^4^	6	The bioinspired, double network hydrogel (BDNH) degraded appropriately and facilitated the formation of neocartilage at the defect site in the animal model. Particularly noteworthy was its thermosensitive nature, which led to enhanced stiffening at physiological temperatures upon implantation.	N/A—histology and immunohistochemistry, rheological testing	18
StarPEG PCL	pigs	mini pigs	6 mm × 1 mm	20 × 10^6^	26	The control empty defects had the highest macroscopic ICRS scores compared to the zonal and non-zonal constructs. To this effect, no significant score differences were found between zonal and non-zonal scaffolds.	**ICRS** Gross Morphology Scores at 26 weeks: Control = ~10 Zonal = ~4 Non-zonal = ~5 (averaged amongst 3 blinded independent scorers)	19
StarPEG vinyl sulfone (sPEG-VS)	mice	mice	1 mm × 1 mm	1 × 10^5^	6, 12	Emphasis on enhancing the mechanical strength of the hydrogels facilitated an optimal environment for cell proliferation. Within the defect model, the development of new hyaline cartilage underscored the scaffold’s biocompatibility, suggesting its potential for future regenerative applications.	**Young’s modulus** 0 weeks = ~1 kPa 1 week = ~6 kPa 3 weeks = ~11 kPa 6 weeks = ~16 kPa 12 weeks = ~30 kPa	20
N-butanol and different concentrations of HA, COL1, B-TCP	rabbits	rabbits	5 mm diameter (full thickness)	5–7 × 10^5^	17, 19	Various experimental groups displayed accelerated rates of cartilage formation compared to the negative control. Among them, TCP + mixed cells + 14 wt% RSF solution and TCP + mixed cells achieved the highest scores. The former maintained a steady score of 11 at 19 weeks, while the latter saw a significant increase in score from approximately 7 to 10 between 17 and 19 weeks.	**ICRS** Gross Morphology Scores at 17 weeks: Blank = 2, A = ~2.5, B = 7, C = 5, D = ~7, E = ~10 Gross Morphology Scores at 19 weeks: Blank = 5, A = 5, B = 6, C = 6, D = 10, E = 11 **KEY: A** = all chondrocytes, **B** = all BMSCs, **C** = mixed cells, **D** = mixed cells + beta-TCP, **E** = mixed cells + beta-TCP + 14 wt% RSF soln.	21
PCL, PLA, PLGA (85:15) and PLGA (65:35)	goats	goats	6 mm × 6 mm	40 × 10^6^ cells/mL of a 3:1 co-culture of FPSCs/chondrocytes	26	Across various categories, there were no significant differences in scores between controls and bi-phasic SA tissues. Nonetheless, in specific categories like matrix staining and cell morphology, distinct significant differences were observed, indicating instances where bi-phasic scaffolds exhibited signs of improvement in repair outcomes.	**ICRS** Average Histology Scores at 26 weeks: Control = ~40 Bi-phasic SA = ~60	22
Human umbilical cord Wharton’s Jelly, GCS + DF-PEG	rabbits	rabbits	~1.2 mm deep	5 × 10^6^ (hydrogel) 2 × 10^5^ (HUCWJ)	13, 26	The biocompatible nature and availability of HUCWJ resulted in satisfactory outcomes post-implantation. Histological analysis revealed effective integration and notable progress in regeneration at both 3 and 6 months. The authors emphasized that extending the incubation period, particularly up to 6 months, greatly reinforces the efficacy of this approach in cartilage regeneration efforts.	N/A—Wakitani Histological Scoring	23

## Data Availability

Not applicable.
